# Enrichment of soil organic carbon by native earthworms in a patch of tropical soil, Kerala, India: First report

**DOI:** 10.1038/s41598-018-24086-8

**Published:** 2018-04-10

**Authors:** S. N. Sruthi, E. V. Ramasamy

**Affiliations:** School of Environmental Sciences, Mahatma Gandhi University, Kottayam, Kerala 686560 India

## Abstract

The role of earthworms in soil carbon dynamics is a recent avenue of research which is less studied in India. Three plots of 1 m^3^ size were laid in Jeevaka live laboratory (JLL)- a biodiversity rich area within the University campus. A control plot (CP) of same dimension was maintained outside JLL. Out of three plots within JLL, one was operated with native earthworm *Perionyx ceylanensis*Michaelson (100 numbers), water and cattle dung as feed (Jeevaka test plot- JT) and fenced with nylon mesh. Remaining two plots were operated as controls within JLL (JC1 and JC2). JC1 (Jeevaka control 1) was provided with cattle dung and water, while JC2 and CP (outside JLL) were operated without any supplements. Throughout the experiment native earthworm species have maintained their dominancy in all plots except CP where no earthworms were observed. At the end of a year-long study, JC1 with maximum diversity of earthworms showed better soil organic carbon (SOC) and particulate organic carbon (POC)-which is relatively a stable form of SOC. Overall findings indicate better the diversity of earthworms better is the carbon storage in the soil.

## Introduction

Earthworms are known for their ecosystem services. Their role in mitigating the global climate change through the soil carbon storage and stabilization is a recent area of research being focused globally^1^. Literature on soil carbon clearly point out the potential of earthworms in influencing carbon and nitrogen dynamics with implications of long-term sustainability and greenhouse gas mitigation in agricultural systems^2,3^. Earthworms are more beneficial to organic farming, where large quantities of carbon based amendments are being used while conventional agriculture relies heavily on mineral fertilizers.

Literature abounds with reports on soil enrichment with organic carbon and nutrients through the interaction of microbes and earthworms^4^. Besides earthworms, termites and other macro fauna do have an inevitable role on soil carbon dynamics through their interaction with microbes. Such interactions are the key factors influencing soil organic carbon (SOC) and help in the dynamics of different fractions of carbon in the soil^5^. Among these fractions, recalcitrant organic carbon (ROC) and particulate organic carbon (POC) are the most stable forms remain in the soil for a decade to century^6^.

In the context of global climate change, soil carbon dynamics becomes an important area of research. Studies on the role of earthworms in soil carbon have been widely reported from many parts of the world^2,7,8^. To date, no study on the role of earthworms in soil carbon dynamics has been reported in India. Hence, the present study was conducted for 12 months in four plots with and without the supplementation of earthworms, feed and water. The focus of this study was to assess how the presence and absence of earthworms in soil affects the carbon dynamics. This study also aims to find out whether supplementation of the experimental plot with a native earthworm species *Perionyx ceylanensis* has any impact on the soil carbon dynamics.

## Materials and Methods

Adult earthworm species *Perionyx ceylanensis* Michaelsen, from the culture being maintained in the Vermitechnology laboratory of the department has been used in this study wherever earthworm supplementation has been carried out.

### Study Area

**Figure 1:**
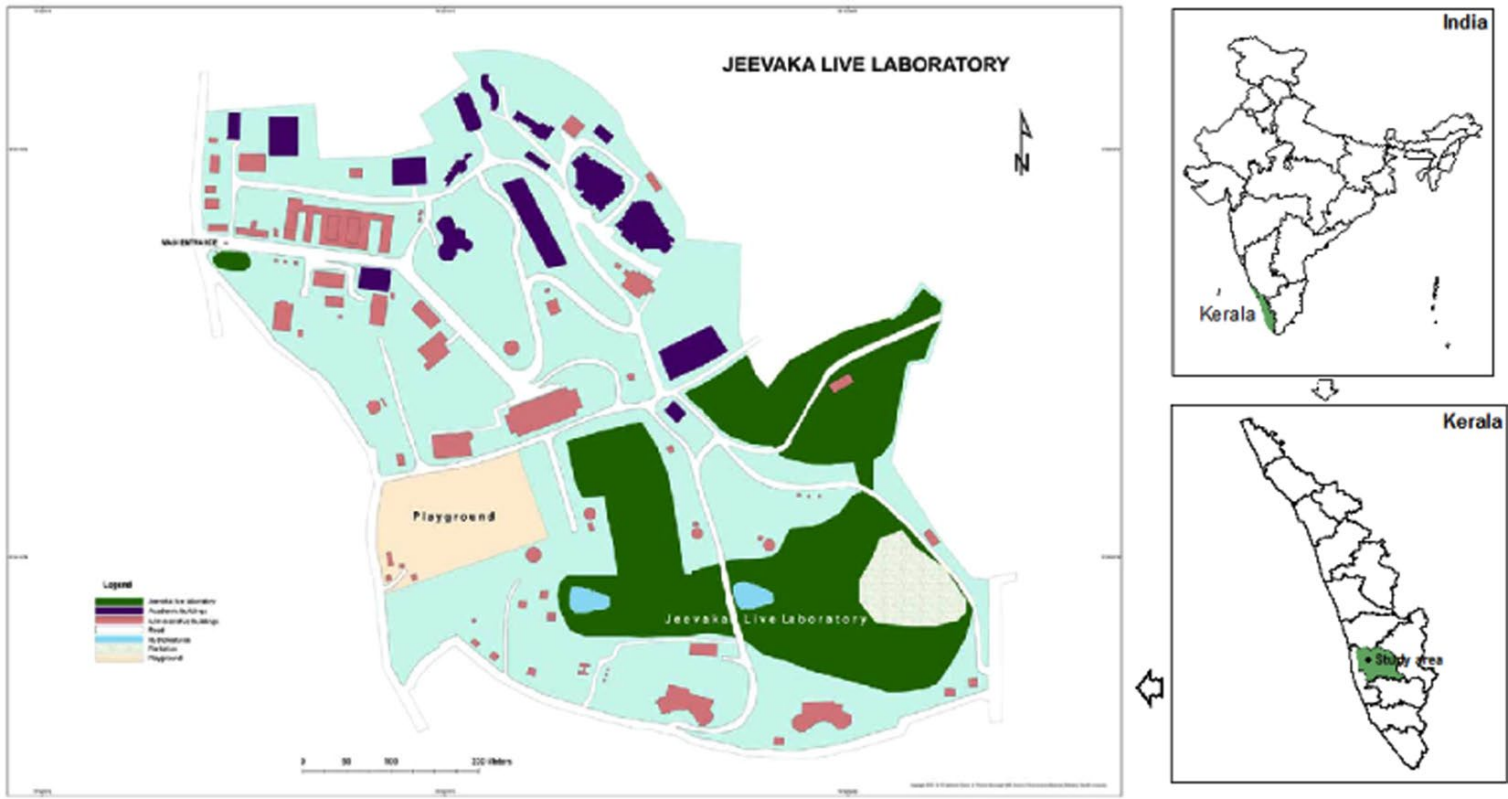
Map showing the study area-Jeevaka live laboratory (Q GIS Version 2.18.14 -new LTR; https://www.qgis.org/en/site/).

Jeevaka live laboratory (JLL), biodiversity rich area within the University campus having natural heterogenic vegetation and least human interference was chosen for this study (Fig. 1). Three plots of size 1 × 1 m were laid within the JLL. One plot of the same dimension was laid outside the JLL (an area with more human interference) as a control.

### Experimental method

Prior to starting of the experiment, earthworm diversity of the study area was assessed. Out of the three plots maintained within the JLL, one was started with an addition of 100 native earthworms (*P. ceylanensis*), water and cattle dung as feed (Jeevaka test plot- JT). This plot was fenced with three layered nylon mesh to a depth of 1 m on all four sides in order to restrict the migration of earthworms between the plot and surrounding area. Other two plots were operated without any fencing and no addition of *P. ceylanensis*.However one was provided with cattle dung as feed (Jeevaka control 1- JC1) and the other one was operated without the feed supplement (Jeevaka control 2- JC2). The control plot outside Jeevaka (CP), located near playground was operated as such, neither earthworms (*P. ceylanensis*) nor any amendments were added. Cow dung (375 g dry weight) was used as feed in JT and JC1 while JC2 and CP were not fed. Feeding was done once in every 30 days. The sprinkling of water was done in JT and JC1 in order to maintain the required soil moisture^9^. Change in soil carbon pool has been monitored throughout the study period by analyzing the soil samples periodically (from all four plots).

### Sampling

Soil samples in triplicates of 10 cm^3^ were collected once in three months from each plot. Soil samples were air dried, ground and sieved prior to analysis.

### Analytical method

Samples were analyzed for the following fractions of soil carbon: Soil Organic Carbon- SOC by Walkley and Black method^10^. Other fractions of SOC such as labile organic carbon-LOC^11^, particulate organic carbon- POC^12^, microbial biomass carbon-MBC^13^ and potential carbon mineralization –PCM^14^ were also analyzed. Prior to start of the experiment, soil samples from all the plots were drawn and analyzed for texture, pH, moisture content, organic carbon (including all fractions of carbon), total organic matter, total nitrogen, available phosphorus and exchangeable potassium as per the procedure of Maiti^15^.

The carbon pool of a given soil is subject to change over a period of time. The changes are due to alterations/conversions occuring in various carbon fractions in the soil. However more prominent changes in the carbon pool are brought by alterations in the labile carbon fraction of the soil. Changes in labile carbon and total organic carbon in soil influence the carbon management index and carbon pool index. Rate of carbon loss and its index have been calculated with lability of carbon (C_L_) and lability index (LI) respectively. Inorder to compare the changes between the labile carbon and total carbon content of the soil, carbon management index (CMI) was calculted. Carbon loss from an ecosystem negatively affect the system, ie; carbon loss from an ecosystem having large carbon pool have less effect than the carbon loss from a system with low carbon pool. Hence, carbon pool index (CPI) was also estimated to assess the changes in carbon pool content of an ecosystem. In this context, to assess the changes in the soil carbon pool the following indices have been calculated as per the equations^16^ given below:

$$\begin{array}{lcc}{\rm{Lability}}\,{\rm{of}}\,\mathrm{carbon}(\mathrm{mg}\,{\rm{labile}}\,{\rm{Carbon}}/{\rm{g}}\,{\rm{soil}}) & = & {\rm{Carbon}}\,{\rm{fraction}}\,{\rm{oxidized}}\,{\rm{by}}\,{{\rm{KMnO}}}^{{\rm{4}}}/{\rm{Carbon}}\\  &  & {\rm{remaining}}\,{\rm{unoxidised}}\,{\rm{by}}\,{\rm{KMnO4}}\end{array}$$

$${\rm{Lability}}\,{\rm{index}}\,({\rm{LI}})={\rm{Lability}}\,{\rm{of}}\,{\rm{carbon}}\,{\rm{in}}\,{\rm{soil}}\,{\rm{sample}}/{\rm{Lability}}\,{\rm{of}}\,{\rm{carbon}}\,{\rm{in}}\,{\rm{reference}}\,{\rm{soil}}$$

$${\rm{Carbon}}\,{\rm{pool}}\,{\rm{index}}\,(\mathrm{CPI})\,{\rm{in}}\,{\rm{mg}}/{\rm{g}}\,=\,{\rm{Total}}\,{\rm{carbon}}\,{\rm{of}}\,{\rm{the}}\,{\rm{soil}}/{\rm{total}}\,{\rm{carbon}}\,{\rm{of}}\,{\rm{the}}\,{\rm{reference}}\,{\rm{soil}}$$

$${\rm{Carbon}}\,{\rm{management}}\,{\rm{index}}\,(\mathrm{CMI})={\rm{CPI}}\times {\rm{LI}}\times {\rm{100}}$$

### Statistical analysis

Two way analysis of variance (ANOVA, p < 0.05), was applied to test the variation in the organic carbon content of the soil from different plots at different time intervals. LSD (Least Significant Difference) was applied in order to determine the level of significance^17^. Linear trend analysis (regression) was also carried out to predict the changes in organic carbon content of the soils.

## Result and Discussion

### Nutrient status of the soil

The changes in nutrient status (N, P, and K), pH and carbon pool in all the plots were determined by analyzing the soil samples collected at zero hour (Table S1) and at the end of the experiment (Table S2). The tri-monthly sampling was done to trace the changes in different fractions of SOC. An increase in total nitrogen and available phosphorus was recorded at the end of the experiment (in all plots) while potassium remained almost same in the course of the yearlong study. The details on the soil texture are given as supplementary table (Table S2).

### Soil organic carbon and organic matter

**Figure 2:**
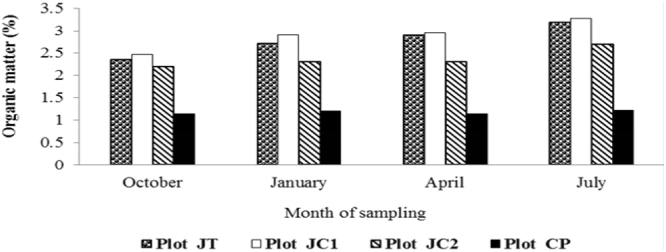
Soil Organic Matter (SOM) content (%) of the samples from different plots.

A gradual increase in the SOM content was recorded in all except the control plot (Fig. 2). However, the increase was not even, JC1 plot showed maximum increase in SOM followed by JT and JC2 and CP.

**Figure 3:**
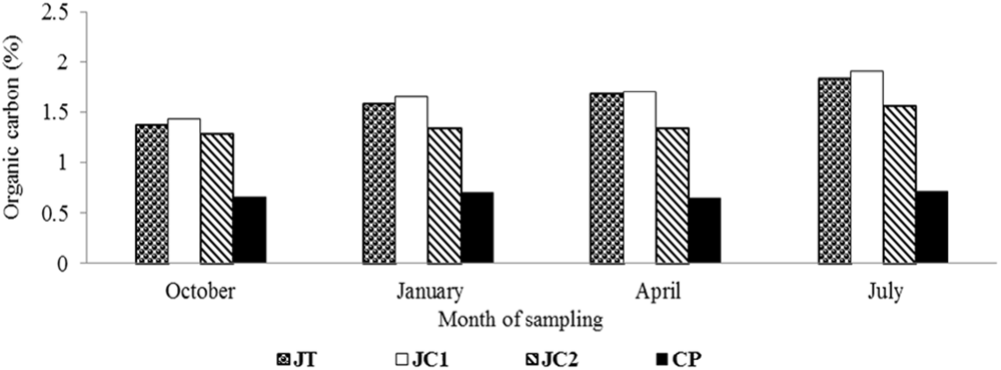
Soil Organic carbon (SOC) content (%) in the soil samples.

**Figure 4:**
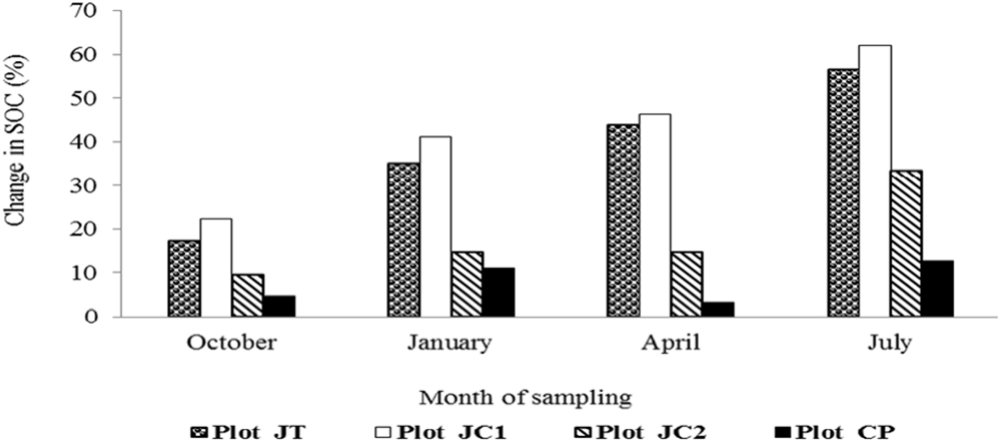
Changes observed in the SOC (%) of the study area.

Soil Organic carbon (SOC) content showed an increasing trend in all except the control plot throughout the experiment (Fig. 3). Similar to SOM, SOC was also found maximum in JC1 (1.91%), followed by JT (1.83%) and JC2 (1.56%) while CP was recorded with minimum (0.71%). In summary, at the end of the experiment, the rate of increase in SOC was 62% in JC1; 56.4% in JT; 33.3% in JC2 and 12.7% in CP (Fig. 4).

As per the results of earthworm diversity assessment done prior to the experiment, the earthworm species *Megascolex konkanensis* Fadarb was observed from all the sampling plots, while *P. corethrurus* showed their presence in only one sampling plot (JC2) of JLL. Increase in soil organic matter noted in these plots could be attributed to the litter degradation enhanced by the earthworm activities^18^. The JC1 plot was recorded with better carbon storage in terms of SOM and SOC than rest of the plots may be due to the presence of more number and diversity of earthworms. Even though JT was also amended with cattle dung and water, the three layered nylon mesh lining placed around the plot might have prevented the entry of earthworms from the surrounding area. Thus, majority of earthworms present in JT were the introduced ones (100 numbers of *Perionyx ceylanensis *Michaelsen). Additionally a few *Megascolex konkenensis* Fadarb recorded in JT plot might have been existing or hatched from the cocoons during the course of the experiment. On the other hand JC1 plot without nylon fence, supplemented with cattle dung and water has attracted variety of earthworms into this plot. Four different species (*Megascolex konkanensis* Fadarb, *Drawida ghatensis* Michaelsen, *Pontoscolex corethrurus* Muller, *and Drawida sp*.) of earthworms were isolated from JC1 at the end of the experiment (Table 1). The interaction of this diverse group of earthworms (Table S3) and soil microbial community might have contributed more SOC and SOM to the soil. Such interaction enhances not only SOC but also nutrients^4^.

**Table 1:** Earthworm population in the study area (After the 12 months of experiment).

Parameters	*M. konkanensis*			*P. corethrurus*			*P. ceylanensis*			*D. ghatensis*			*Drawida sp*.		
	JT	JC1	JC2	JT	JC1	JC2	JT	JC1	JC2	JT	JC1	JC2	JT	JC1	JC2
Frequency	100	100	100	0	100	0	100	0	0	0	100	67	0	66.7	0
Density	2	4	1	0	3.34	0	2.67	0	0	0	2.0	7	0	1	0
Abundance	3	4	1.5	0	3.34	0	2.67	0	0	0	2.0	1.5	0	1.5	0
Relative Density	35.4	38.7	100	0	32.3	0	64.6	0	0	0	19.4	10.1	0	9.7	0

Earthworm activities besides enhancing the soil carbon content also store more carbon in their burrows and cast^8^. It is also reported that the organic carbon present in the earthworm casts are well protected^7,19,20^.

### Different fractions of soil organic carbon (SOC)

Among the soil carbon fractions, particulate organic carbon (POC) is relatively stable and is considered as a sensitive indicator of soil carbon management^12^. Microbial biomass carbon (MBC) generated mainly through the microbial activity is a fraction which responds rapidly to the physical changes in the soil, hence it is less stable^3^. Labile organic carbon (LOC) is yet another fraction of SOC having rapid turnover rate on oxidation, it releases CO_2_^21^.

#### Particulate organic carbon

POC is an important fraction of soil organic carbon as it is relatively stable and remains in the soil for a decade or even more^6^. Hence the level of POC and its dynamics is given more importance in soil carbon studies.

At the end of the 12 month study, higher level of POC was recorded in JC1 (0.69% ± 0.11) followed by JT with 0.65% ± 0.17 and JC2 with 0.56% ± 0.11 (Table 2). JC1 showed a maximum increase of 50% in POC with respect to the initial stage of the experiment, while JT and JC2 were recorded with 35.4% and 21.7% increase in POC respectively. The plot CP was recorded with only 9.1% increase in POC content. This trend again indicates that the presence of more (diverse) earthworm species influence POC. Rich POC content in plots with diverse species of earthworms confirms their role in storing more stable form of soil carbon.

**Table 2:** Different fractions of SOC (%) observed in the study area.

Months	Sample Plots											
	JT			JC1			JC2			CP		
	MBC	LOC	POC	MBC	LOC	POC	MBC	LOC	POC	MBC	LOC	POC
July (Zero hour)	0.18 ± 0.06	0.31 ± 0.02	0.48 ± 0.08	0.16 ± 0.02	0.31 ± 0.07	0.46 ± 0.05	0.17 ± 0.04	0.32 ± 0.08	0.46 ± 0.12	0.09 ± 0.03	0.20 ± 0.04	0.22 ± 0.06
October	0.22 ± 0.03	0.33 ± 0.12	0.54 ± 0.14	0.20 ± 0.08	0.33 ± 0.04	0.55 ± 0.12	0.17 ± 0.07	0.35 ± 0.07	0.51 ± 0.13	0.10 ± 0.02	0.21 ± 0.04	0.23 ± 0.07
January	0.29 ± 0.12	0.37 ± 0.10	0.61 ± 0.14	0.30 ± 0.09	0.36 ± 0.10	0.63 ± 0.15	0.25 ± 0.07	0.41 ± 0.09	0.53 ± 0.13	0.11 ± 0.05	0.22 ± 0.11	0.23 ± 0.04
April	0.33 ± 0.09	0.41 ± 0.11	0.61 ± 0.14	0.35 ± 0.11	0.40 ± 0.06	0.64 ± 0.09	0.28 ± 0.04	0.39 ± 0.07	0.51 ± 0.13	0.10 ± 0.03	0.20 ± 0.10	0.23 ± 0.09
July	0.34 ± 0.12	0.41 ± 0.12	0.65 ± 0.17	0.36 ± 0.14	0.42 ± 0.10	0.69 ± 0.11	0.29 ± 0.04	0.43 ± 0.07	0.56 ± 0.11	0.12 ± 0.06	0.22 ± 0.09	0.24 ± 0.11

JT-Jeevaka Test, JC1-Jeevaka Control 1, JC2-Jeevaka Control 2 and CP- Control Plot (outside JLL).

Earthworms positively influence the formation of POC and protect the same within micro and macro-aggregates^22^. Present study also recorded an enhancement of 0.66 and 0.18 times of more POC in the plots with and without earthworms during the entire period. Similar observations of POC increments in the soils with earthworms have been reported by Bossuyt *et al*.^7^.

#### Microbial biomass carbon

MBC level has exhibited an increasing trend in all plots during the experiment *albeit* unevenly. JC1 has recorded the highest MBC of 0.36% ± 0.14 while CP had only 0.12% ± 0.06 (Table 2). The percentage increase in MBC was highest (125%) in JC1 followed by JT (88.9%) and JC2 (68.1%). The control plot (CP) showed only 20% increase in MBC. Earlier studies have reported an increase of 1.4 fold MBC in the soil with earthworms compared to the soil without earthworms^23^. However,the findings of the current study shows 1.05 fold increase in MBC (JC1) compared to CP. MBC is a resultant of microbial activity in the soil which inturn is influenced by the diverse earthworm species in the soil^23^.

Different strata of microbial consortia and their activity could enrich the soil with more carbon than a single group can. This has been reflected in MBC, thus JC1 showed more MBC than the rest of the plots.

#### Labile organic carbon

Overall increase in LOC was recorded at the end of the experiment in all the plots with fluctuations (Table 2). The variation in LOC among the plots within JLL was insignificant (0.01%) with JC2 on the top follow by JC1 and JT. CP remained with least level of LOC. LOC content of the soil mainly depends on the litter deposition and microbial degradation^24^, as both these criteria are met within JLL the increase in LOC was more in plots inside JLL than the CP.

#### Potential Carbon Mineralization (PCM)

Potential carbon mineralization is an indicator of soil microbial activity in mineralizing the carbon content of the soil. At the first quarter of this experiment around 4.9%, carbon mineralization was recorded in JT plot and 12.2% in JC1. Eventhough the plot with more number of earthworms is expected to show more carbon mineralization^3^ it was not reflected prominently in present study. For instance JC1 had more earthworms (Table S2) compared to JT, however the difference in mineralization was hardly 3% towards the end of the experiment. The carbon mineralization recorded in all of the study showed the following trend: JC1 > JT > JC2 > CP.

### Carbon turnover

The carbon content of the soil mainly depends on the size of total carbon pool and the rate of carbon turnover. Carbon turnover in terms of the lability of carbon, lability index (LI), carbon pool index (CPI) and carbon management index (CMI) were calculated in this study and the results are given in Table 3. After the completion of the experiment, in JT and JC1 plots the labile carbon fractions remained same (0.22) so also the lability index (0.71). Thus the carbon turnover rate also remained same in both plots.

**Table 3:** Result of carbon turnover analysis.

Soil samples (Period of study)	Carbon analysis			
	Lability of carbon (mg C/g soil)	Lability Index (LI)	Carbon Pool Index (CPI) (mg/g)	Carbon Management Index (CMI)
JT sample(One year)	0.22	0.71	1.17	83.1
JC1 sample(One year)	0.22	0.71	1.22	86.6

JT-Jeevaka Test, JC1-Jeevaka Control1.

The loss of carbon from a unit area with lesser quantity of carbon pool cause more concern than from an area with large carbon pool because, calculations of CPI incorporates both loss of carbon and the carbon pool of an area. The results of this study showed JC1 plot with high carbon pool index (1.22) than JT (1.17). Thus the loss of carbon affects more JT than JC1. The carbon management of a system is better expressed in terms of carbon management index (CMI). From the results of one year long study, the JC1 plot showed CMI of 86.6 where as that of JT was 83.1. In summary, JC1 had better carbon management system than JT^16^.

Among the plots maintained with in JLL, JC1 was observed with high POC, MBC, PCM and total carbon pool. Even though an increase in total carbon pool was noted in all the plots during the course of this experiment, JC1 was recorded with maximum of organic carbon, MBC and with high rate of carbon mineralization. Occurrence of high MBC and high rate of mineralisation in JC1 could be attributed to the diverse microbial population and their activities. Whereas, high concentration of POC in JC1 could be due to the microbial activity as well as the formation of micro-macro aggregates by earthworms and microorganisms together.

JC1 plot with better CMI indicates better storage of soil organic carbon despite of the fact that, the PCM of this plot is higher than JT. In spite of higher mineralization in JC1, the carbon storage in the form of POC is also better in this plot mainly because of the combined activity of microbes and earthworms^25^.

In summary, the JC1 plot with better diversity of earthworms (*M. konkanensis*, *D. ghatensis*, *P. corethrurus*, *Drawida sp*.) and majority of earthworms are being native species (87% are native and 16% are exotic) which in turn influence better microbial activity as indicated by high MBC and high PCM values observed with higher POC content. While, the JT plot was observed with two native earthworms (*M. konkanensis* and *P. ceylanensis*) and JC2 also with two species of native earthworm (*M. konkanensis* and *D. ghatensis*). Whereas the control plots CP (outside Jeevaka) had no earthworms in it .

### Statistical analysis

The results of the ANOVA indicate a significant difference (F = 102.39) in organic carbon content of the soil from different plots. Similar trend is observed between the soil samples collected from the plots at different time intervals (F = 8.34) of the experimental tenure (p < 0.05). The LSD analysis also reveals that JC1 is with most significant level of difference (best performing plot) in SOC concentration among the rest of the plots. Linear trend analysis result also infers that the SOC from all plots exhibit an increasing trend, which is more prominent in JC1 and JT plots.

## Conclusion

The findings of this study leads to the following conclusions:

1. Earthworms play an important role in soil carbon dynamics.
2. The natural ecosystem existing in JLL has led to better soil carbon dynamics.
3. The plot with more diverse earthworm species (JC1) has performed better than the plots with lesser diversity of earthworms. The diversity of earthworms influences the microbial activity in soil which in turn results in better carbon pool.
4. Availability of feed and water enhances the diversity of earthworms which in turn improve the soil carbon pool as observed in the JC1.
5. The high values of CPI (carbon pool index) in JC1 revealed the existence of all forms of carbon in the soil. This further indicates a better carbon management system prevailing in JC1 thus the CMI (carbon management index) of JC1 is also high.

## Electronic supplementary material

Supplementary materials
