# Supplementary material for: Enrichment of soil organic carbon by native earthworms in a patch of tropical soil, Kerala, India: First report
Source: Sci Rep. 2018 Apr 10;8:5784. doi: 10.1038/s41598-018-24086-8 (PMC5893537; doi:10.1038/s41598-018-24086-8)
Supplement: Supplementary file 1 — Supplementary materials [file 41598_2018_24086_MOESM1_ESM.docx]

**Enrichment of soil organic carbon by native earthworms in a patch of tropical soil, Kerala, India: First report**

**Sruthi S N and E.V.Ramasamy^*^**

*Corresponding Author e-mail: [evramasamy@](mailto:evramasamy@)mgu.ac.in

School of Environmental Sciences, Mahatma Gandhi University, Kottayam, Kerala 686560, India. Mobile: + 91 9447095935

**Supplementary Material**

| Sl.No | Soil Sample | pH | Texture | Total Nitrogen (%) | Available Phosphorous (%) | Exchangeable potassium (%) |
| --- | --- | --- | --- | --- | --- | --- |
| 1 | JCT | 4.36 ±1.94 | Loamy sand | 2.0 ± 0.79 | 0.06 ± 0.014 | 0.40 ± 0.01 |
| 2 | JC1 | 4.60 ±0.37 | Loamy sand | 2.20± 0.93 | 0.05 ± 0.02 | 0.38 ± 0.04 |
| 3 | JC2 | 4.53± 1.21 | Loamy sand | 1.9 ± 0.64 | 0.06 ± 0.01 | 0.42 ± 0.03 |
| 4 | CP | 6.62 ±1.24 | Sandy clay loam | 0.9 ± 0.25 | 0.02 ± 0.008 | 0.15 ± 0.01 |

Table S1: Nutrient status of soil at zero hour

JT-Jeevaka Test, JC1-Jeevaka Control 1, JC2-Jeevaka Control 2 and CP- Control Plot (outside)

Table S2: Nutrient status of soil at the end of the experiment (after one year)

| Soil Sample | pH | Texture | Total Nitrogen % | Available Phosphorous (%) | Exchangeable potassium (%) |
| --- | --- | --- | --- | --- | --- |
| JT | 5.4± 1.2 | Loamy sand | 2.9± 1.3 | 1.1 ± 0.40 | 0.44 ± 0.12 |
| JC1 | 5.8± 0.76 | Loamy sand | 3.2± 0.94 | 1.4 ± 0.37 | 0.47 ± 0.23 |
| JC2 | 6.9± 1.53 | Loamy sand | 2.3±0.76 | 0.08 ± 0.03 | 0.43 ± 0.14 |
| CP | 6.7± 1.45 | Sandy clay loam | 1.1± 0.56 | 0.03 ± 0.01 | 0.16 ± 0.08 |

| Quadrates | JT plot | | JC1 plot | | | | JC2 plot | |
| --- | --- | --- | --- | --- | --- | --- | --- | --- |
|  | *P.ceylanensis* | *M.konkanensis* | *M.konkanensis* | *P. corethrurus* | *D.ghatensis* | *D sp.* | *M.konkanensis* | *D.ghatensis* |
| I | 5 | 3 | 3 | 2 | 4 | 2 | 2 | 2 |
| II | 7 | 4 | 2 | 4 | 3 | 2 | 1 | 2 |
| III | 6 | 4 | 5 | 1 | 4 | 0 | 3 | 0 |
| IV | 8 | 0 | 2 | 6 | 5 | 0 | 2 | 4 |
| V | 10 | 2 | 6 | 3 | 0 | 2 | 2 | 3 |
| Total No. of worms/plot | **49** | | **56** | | | | **21** | |

Table S3: Total number of earthworms observed through the quadrate study after the completion of the experiment

*P.ceylanensis : Perionyx ceylanensis*

*M. konkanensis : Megascolex konkanensis*

*P.corethrurus : Pontoscolex corethrurus*

*D.ghatensis : Drawida ghatensis*

*D sp. : Drawida species*
